# Great saphenous vein aneurysm mimicking inguinal hernia: a case report

**DOI:** 10.1186/s13256-022-03655-6

**Published:** 2022-11-10

**Authors:** Hamed Ghoddusi Johari, Hamidreza Malekhosseini, Amirhossein Erfani, Reza Shahriarirad

**Affiliations:** 1 Trauma Research Center, Vascular Surgery Department, Shiraz University of Medical Sciences, Shiraz, Iran; 2 Thoracic and Vascular Surgery Research Center, Shiraz University of Medical Sciences, Shiraz, Iran; 3 Student Research Committee, Shiraz University of Medical Sciences, Shiraz, Iran

**Keywords:** Inguinal hernia, Great saphenous vein aneurysm, Misdiagnosis, Aneurysm

## Abstract

**Background:**

Diagnosis of venous aneurysm may be difficult and can be misjudged as mass-like lesions, including hernias. Here we present the case of a patient with a great saphenous vein aneurysm misdiagnosed and operated as an inguinal hernia.

**Case presentation:**

A 39-year-old Middle Eastern/Persian male presented with left inguinal bulging 15 years ago, which was misdiagnosed and operated on with a diagnosis of inguinal hernia. He was referred to our clinic, in which color Doppler sonography revealed left-sided saphenofemoral junction incompetence with severe flow reversal during the Valsalva maneuver, in favor of a great saphenous vein aneurysm. Ligation of left saphenofemoral junction and stripping of saphenous vein, and stab avulsion phlebotomy of left lower extremity varicose veins, were done. He was discharged the next day after the operation with an uneventful postoperative course or complication on follow-up.

**Conclusion:**

Venous aneurysms can be misdiagnosed as other, more common, mass-like lesions, such as inguinal hernias. Therefore, our report emphasizes the consideration of thorough assessment and utilization of color duplex sonography to prevent further misdiagnosis and unnecessary intervention and operations.

## Introduction

A venous aneurysm is defined as a localized dilatation of a vein that connects to the main vein structure through a single channel. Venous aneurysms are unusual vascular pathologies that often appear as asymptomatic and incidental findings on imaging studies or physical examinations [1]. Yet, they may present symptoms such as pain or mass-like lesions depending on the location of the pathology. Venous aneurysms can present anywhere throughout the venous system; however, they are more common in lower extremities, especially in deep venous systems. Diagnosis of venous aneurysms may be difficult and can be mistaken for mass-like lesions, including hernias [1]. Here we present the case of a patient with a great saphenous vein aneurysm misdiagnosed and operated as an inguinal hernia.

## Case presentation

A 39-year-old Middle Eastern/Persian male with a known case of left lower extremity varicose vein was referred to our vascular surgery service with a chief complaint of left inguinal bulging since 15 years ago. He was asymptomatic and reported no pain or restrictions in movement or other clinical findings aside from the bulging, which resolved under local pressure. The patient had been misdiagnosed and operated on 10 years ago with the impression of a left inguinal hernia; however, no hernia sac was detected at that time.

**Fig. 1:**
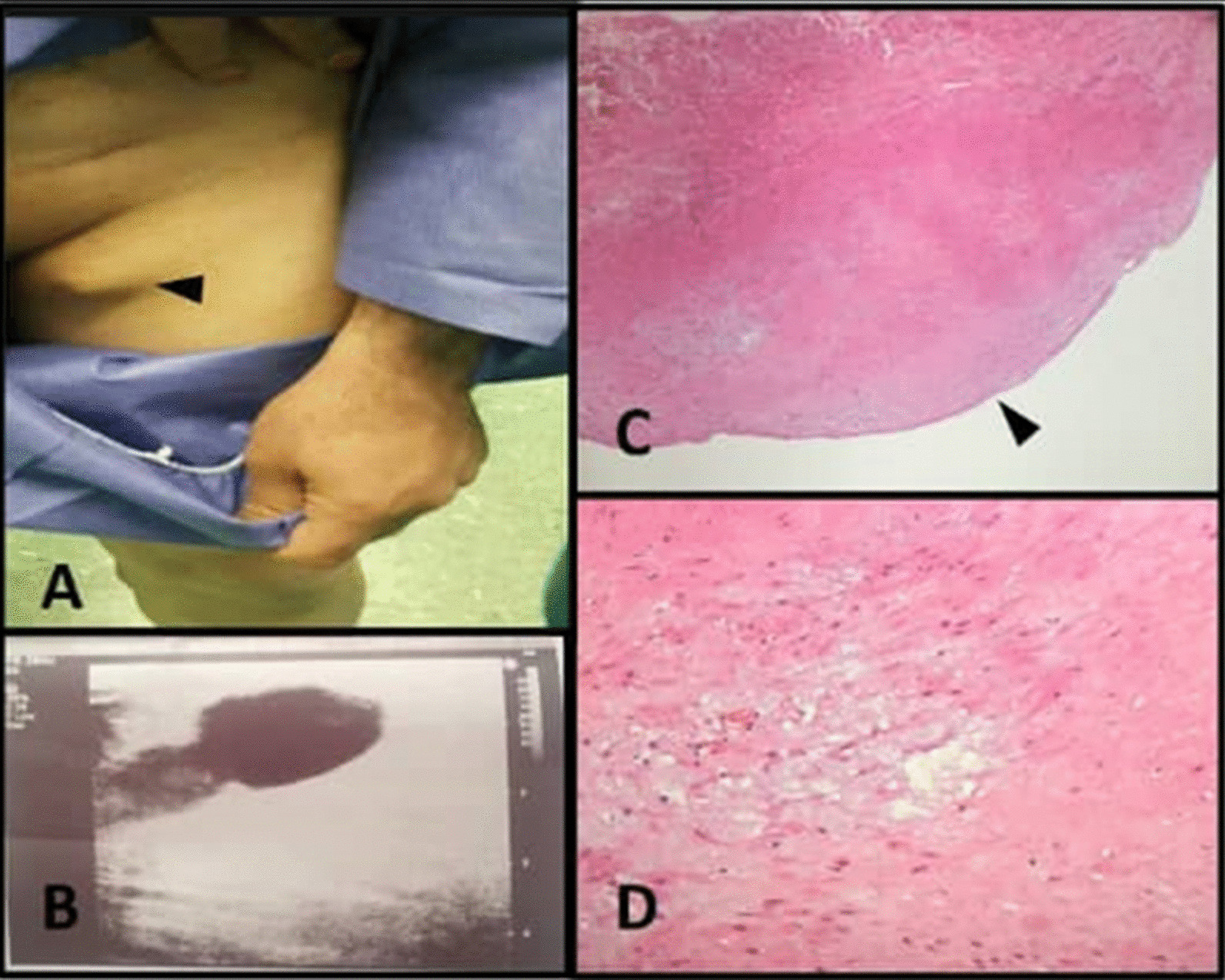
**A** Soft, nonpulsatile, reducible inguinal bulging in the left inguinal area; **B** color Doppler sonography of the left lower extremity venous system revealed incompetency of the left-sided saphenofemoral junction and a large venous aneurysm originating from the left-sided saphenofemoral junction; **C** histopathology section of the aneurysmal lesion demonstrating dilated uniloculated mass with endothelial lining, indicative of tunica intima (arrowhead) (×100 hematoxylin and eosin stain); **D** the lesion’s wall cut section demonstrating thickening of the three layers of the venous histology with destruction of tunica media and its replacement by loose connective tissue with mild myxoid degeneration (×400, hematoxylin and eosin stain)

Five years later, the patient developed left lower extremity varicose veins. On physical examination, severe varicose veins of the left lower extremity with a soft, nonpulsatile, reducible inguinal bulging were detected that became more prominent by performing the Valsalva maneuver (Fig. 1).

On the basis of the patient features, color Doppler sonography of left lower extremity venous system was performed, which revealed left-sided saphenofemoral junction incompetence with severe flow reversal during the Valsalva maneuver (Fig. 1).

**Fig. 2:**
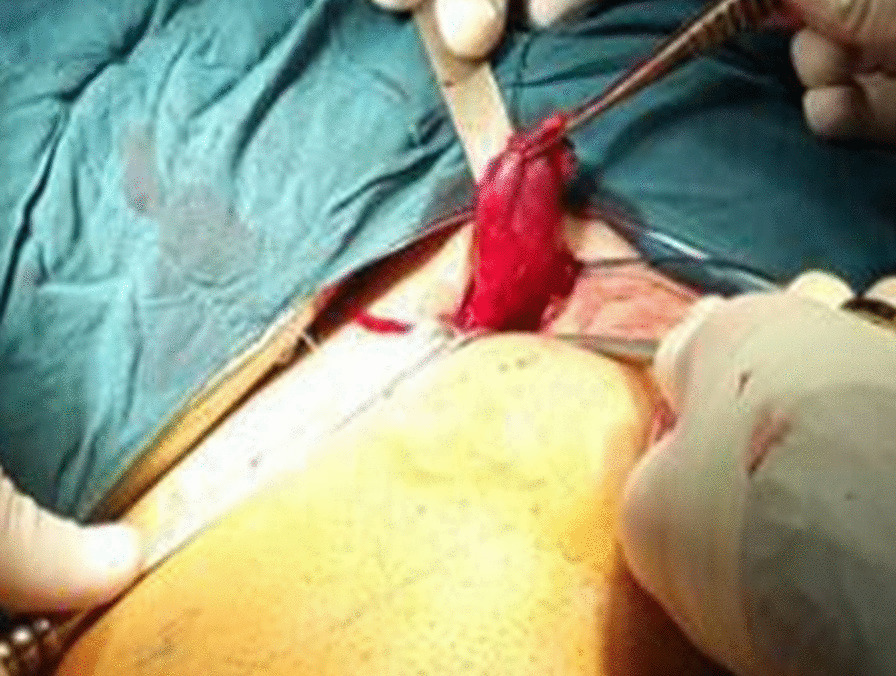
Venous aneurysm of the great saphenous vein with 2 cm distance from the left saphenofemoral junction

The patient was scheduled for surgery in which ligation of the left saphenofemoral junction and stripping of the saphenous vein, and stab avulsion phlebotomy of the left lower extremity varicose veins, were done. In the course of surgery, a 5 × 2 cm venous aneurysm of the great saphenous vein with about 2 cm distance from the saphenofemoral junction was detected. (Fig. 2). Subsequently, the aneurysm was resected and sent for pathological evaluation. (Fig. 1). No complications occurred during the operation. The patient was discharged from the hospital on the next day after operation. After 1 month, the patient visited the clinic with an uneventful postoperative course and without any complaint.

## Discussion

Venous aneurysms are dilatations of veins with single communication to the main venous system without any association with pseudoaneurysm or arteriovenous fistula [2]. They are usually asymptomatic but may present as painful masses or cause complications such as deep vein thrombosis or pulmonary embolism [3]. Patients could be misdiagnosed as having other mass-like lesions, such as in our case, and undergo unnecessary intervention and operations. Therefore, careful and thorough evaluation of these cases are justified.

Previous studies documented the presence of venous aneurysms in different parts of the venous system, such as portal, superior vena cava, intracranial, popliteal, splenic, and great saphenous vein, among which the most common site in the lower extremity is the popliteal vein, followed by the saphenous vein. The distribution of venous aneurysms is the same in both sexes and can occur in any age group. The exact etiology of venous aneurysms is not clearly defined, although trauma, weakness of the venous wall due to a congenital defect in connective tissue structure, degenerative changes, and inflammation can be the basis of aneurysm formation [4, 5].

There are four types of venous aneurysm proposed by Pascarella *et al*.: type I, which is the most common type (52%), is located in the proximal third of the saphenous vein, not at the saphenofemoral junction but instead just distal to the subterminal valve; type II venous aneurysms are located in the distal third of the thigh in the shaft of the saphenous vein; type III is the coexistence of types I and II in the same extremity; type IV is an aneurysm of the superficial small saphenous vein [6].

The differential diagnosis of a mass-like lesion or bulging on the lower extremities with the indication of surgery has a broad spectrum, and misdiagnosis may lead to complications and difficulties for the patient and the surgeon. Nowadays, the noninvasive diagnostic method is color Doppler sonography. Venous disorders can be easily distinguished from other conditions using a color Doppler examination. The diagnosis may also be aided by ultrasonography or venography. Nevertheless, the venographic diagnosis may be hampered by the presence of thrombi within the venous aneurysmal sac. To detect thrombus within the aneurysmal sac and measure the aneurysm's diameter, venous color Doppler ultrasonography is superior to venography [7].

There are several surgical techniques, including ligation, simple excision, excision and vein patching, tangential excision with lateral venography, and complete resection, for the management of venous aneurysms. Endovenous laser, endovenous radiofrequency, and sclerotherapy can be used as an alternative therapy in selected cases [1, 8].

## Conclusion

We reported a case of a venous aneurysm that was resected during stripping of the great saphenous vein. A venous aneurysm can be misdiagnosed as other, more common, mass-like lesions, such as inguinal hernias. This case was misdiagnosed with an inguinal hernia and operated once with the impression of an inguinal hernia; thus, our report emphasizes the consideration of thorough assessment and utilization of color duplex sonography to prevent further misdiagnosis and unnecessary intervention and operations.

## Data Availability

All data regarding this study has been reported in the manuscript. Please contact the corresponding author if you are interested in any further information.
